# Genetic Literacy and Communication of Genetic Information in Families Concerned with Hereditary Breast and Ovarian Cancer: A Cross-Study Comparison in Two Countries and within a Timeframe of More Than 10 Years

**DOI:** 10.3390/cancers13246254

**Published:** 2021-12-13

**Authors:** Carla Pedrazzani, Chang Ming, Nicole Bürki, Maria Caiata-Zufferey, Pierre O. Chappuis, Debra Duquette, Karl Heinimann, Viola Heinzelmann-Schwarz, Rossella Graffeo-Galbiati, Sofia D. Merajver, Kara J. Milliron, Christian Monnerat, Olivia Pagani, Manuela Rabaglio, Maria C. Katapodi

**Affiliations:** 1 Department of Clinical Research, Faculty of Medicine, University of Basel, 4055 Basel, Switzerland; carla.pedrazzani@unibas.ch (C.P.); chang.ming@unibas.ch (C.M.); 2 Women’s Clinic, University Hospital Basel, 4031 Basel, Switzerland; nicole.buerki@usb.ch (N.B.); viola.heinzelmann@usb.ch (V.H.-S.); 3 Department of Business Economics, Health and Social Care, University of Applied Science and Arts of Southern Switzerland, 6928 Manno, Switzerland; maria.caiata@supsi.ch; 4 Oncogenetics Unit, Service of Oncology, University Hospital of Geneva, 1205 Geneva, Switzerland; Pierre.Chappuis@hcuge.ch; 5 Feinberg School of Medicine, Northwestern University, Chicago, IL 60208, USA; debra.duquette@northwestern.edu; 6 Medical Genetics, University Hospital Basel, 4031 Basel, Switzerland; karl.heinimann@usb.ch; 7 Oncology Institute of Southern Switzerland, 6900 Lugano, Switzerland; rossella.graffeogalbiati@eoc.ch (R.G.-G.); opagani@bluewin.ch (O.P.); 8 University of Michigan School of Public Health, Ann Arbor, MI 48109, USA; smerajve@umich.edu; 9 Roger Comprehensive Cancer Center, University of Michigan, Ann Arbor, MI 48109, USA; kmilliro@med.umich.edu; 10 Jura Hospital, 2800 Delémont, Switzerland; christian.monnerat@h-ju.ch; 11 Inselspital University Hospital, 3010 Bern, Switzerland; manuela.rabaglio@insel.ch

**Keywords:** genetic counselling, family communication, genetic information, informing at-risk relatives, knowledge of genetic risk factors, genetic affinity, sensitivity analysis

## Abstract

**Simple Summary:**

This cross-study comparison uses data collected over 10 years from families living in the US and in Switzerland in order to compare genetic literacy between individuals who had genetic counselling for hereditary breast/ovarian cancer (HBOC) and one or more of their relatives who did not, and examines factors influencing genetic literacy both at the individual and at the family level. The study identifies genetic risk factors and signs of HBOC that remain unclear, even to individuals who had genetic consultation, and highlights the gaps in the dissemination of genetic information. Sensitivity analysis examines the dissemination of genetic information from the individual who had counselling to relatives within the same family that did not.

**Abstract:**

Examining genetic literacy in families concerned with hereditary breast and ovarian cancer (HBOC) helps understand how genetic information is passed on from individuals who had genetic counseling to their at-risk relatives. This cross-study comparison explored genetic literacy both at the individual and the family level using data collected from three sequential studies conducted in the U.S. and Switzerland over ≥10 years. Participants were primarily females, at-risk or confirmed carriers of HBOC-associated pathogenic variants, who had genetic counselling, and ≥1 of their relatives who did not. Fifteen items assessed genetic literacy. Among 1933 individuals from 518 families, 38.5% had genetic counselling and 61.5% did not. Although genetic literacy was higher among participants who had counselling, some risk factors were poorly understood. At the individual level, genetic literacy was associated with having counselling, ≤5 years ago, higher education, and family history of cancer. At the family level, genetic literacy was associated with having counselling, higher education, and a cancer diagnosis. The findings suggest that specific genetic information should be emphasized during consultations, and that at-risk relatives feel less informed about inherited cancer risk, even if information is shared within families. There is a need to increase access to genetic information among at-risk individuals.

## 1. Introduction

Genetic literacy is the ability to understand and use genetic information for health-related decision-making [1,2]. It refers to awareness about genetic risk factors, how they contribute to disease, understanding the chance of inheriting the genetic predisposition and developing the disease [1,2,3,4]. Genetic literacy facilitates seeking genetic evaluation and making informed decisions about genetic testing [1,3,5]. However, there are significant knowledge gaps in the general population, in stark contrast to the current levels of genetic and genomic discoveries and achievements in medicine and public health [1,3,6,7]. Factors like age, race and ethnicity, education and socioeconomic status, and personal and family health history influence genetic literacy [3,6,8,9], as well as access to specialized services [1,10]. Finally, variations in genetic literacy have been reported for people living in different countries [7,9].

Genetic literacy is especially important for families concerned with actionable (Tier 1) genetic conditions, such as hereditary breast and ovarian cancer (HBOC) [11]. HBOC is caused by germline autosomal dominant pathogenic variants; first-, second-, and third-degree relatives have a 50%, 25%, and 12.5% probability, respectively, of inheriting the familial pathogenic variant [12]. In addition to managing the cancer risk of individuals carrying HBOC-associated pathogenic variants, it is also essential to address the potentially increased risk to relatives through cascade testing [11,13]. Due to privacy laws in most countries, individuals carrying HBOC-associated variants have a key role in disseminating genetic information to relatives and in advocating for cascade testing [14,15]. The proportion of relatives who initiate contact with genetic services and their knowledge of cancer genetics increases with genetic consultation [16,17], and when counselled individuals share information received during the consultation process [18,19].

Examining genetic literacy in the context of HBOC helps understand how genetic information is passed on from healthcare providers to index cases i.e., first in the family identified with a pathogenic variant, during genetic counselling, and from index cases to relatives. This is an essential step to support HBOC cascade testing. The purpose of this study is to explore genetic literacy among individuals who had genetic counselling for HBOC, i.e., whether they can recall information about genetic risk factors, modes of inheritance, and probability of developing an HBOC-associated cancer, and how much of this information has been shared with their relatives. Specific aims are first to describe and compare genetic literacy between two groups of individuals, namely those who had genetic counselling for HBOC and their relatives who did not; and second to explore factors influencing genetic literacy both at the individual and at the family level. To achieve these aims we examined data collected from three sequential studies conducted in the U.S. and Switzerland over a timeframe of more than 10 years. Pooling data across studies is feasible, since there are many similarities in the delivery and contents of genetic counselling in different countries [20].

## 2. Materials and Methods

This cross-study comparison used descriptive data from three family-based studies: a cross-sectional study conducted in 2007 in the US [21], baseline data from a randomized trial (RCT) conducted in 2012 in the US (NCT 01612338) [22], and baseline data from an ongoing cohort initiated in 2017 in Switzerland (NCT03124212) [23]. All studies were approved by the appropriate Institutional Review and Scientific Advisory Boards and Ethical Committees (HUM00011707 and HUM00055949, approved on 10 May 2007 and 14 October 2011 respectively, are exempt due to analysis of fully anonymized data; BASEC 2016-02052, approved on 6 February 2017, is ongoing). For this cross-study comparison we pooled participants and divided them into two distinct groups: individuals who had genetic counselling for HBOC, i.e., “expose-d” to counselling and one or more of their first, or second-, or third-degree relatives who did not have counselling, i.e., “not exposed”.

All three studies recruited individuals 18 years or older using the same procedures, identifying potentially eligible participants either from genetic clinics [21,23] or from a state-wide cancer registry [22]. The 2007 US-based cross-sectional study identified females who had genetic counselling in a comprehensive cancer center with approximately 65% identified as carrying an HBOC-associated pathogenic variant [21]. The 2012 US-based RCT identified females diagnosed with breast cancer younger than 45 years old from a state-wide cancer registry, with 25% reportedly receiving genetic consultation at enrolment [22]. The Swiss-based cohort recruits both males and females who are confirmed carriers of an HBOC-associated pathogenic variant and who joined the cohort between January 2017 and January 2021 [23].

In all three studies, potentially eligible participants were mailed study materials from each recruitment site (genetic clinic or cancer registry). Those agreeing to participate returned a signed consent form, revealing their name and address to the research team, and were asked to approach and pass on recruitment materials to relatives. Relatives who accepted participation also returned a signed consent, revealing their name, address, and degree of biological relation to the person who initiated the invitation. Inviting relatives was not a mandatory requirement for participating in the three studies, while each participant could invite one or more relatives. Details about recruitment of participants and relatives have been reported for each original study [21,22,23]. All studies mailed self-administered questionnaires, which were identical for those that had counselling and those who did not.

Genetic literacy was assessed with items used in all three studies, and was conceptualized as having two components, i.e., objective knowledge of cancer genetics and genetic affinity [9,24]. Objective knowledge of cancer genetics included genetic risk factors, and probabilities of carrying a pathogenic variant and developing the disease. This information consists the “core knowledge” explained during genetic counselling. Objective knowledge was assessed with 13 items, asking participants to respond “True”, “False”, or “Do not Know” to statements related to this “core knowledge” [25]. Objective knowledge of cancer genetics was examined first through an overall score, calculated by summing the number of correct answers, and second by examining each knowledge item individually to reveal patterns of potentially not well-understood information. Cronbach’s α was greater than 0.85 in all three original studies and was 0.88 in the whole sample of the cross-study comparison. Genetic affinity, i.e., perceptions of being informed about cancer genetics and cancer risk, was assessed with two items asking: “How well informed do you feel about the probability of getting cancer?” ranging from 1 “Not at all informed” to 7 “Very Informed” and “How much do you know about the genetics of cancer?” ranging from 1 “Not at all” to 7 “A great deal”. A genetic affinity score was calculated by summing responses in these two items.

Questionnaires also assessed demographics i.e., age, gender, race and ethnicity, marital status, education, employment, and clinical characteristics i.e., personal history of cancer “Yes” or “No”; family history of cancer “Yes” or “No”; years since personal cancer diagnosis “≤5 years” or “>5 years”; and years since genetic counselling “≤5 years” or “>5 years”. We selected five years as a cut-off to assess the relevance of personal cancer diagnosis and years since genetic counselling since international guidelines consider this timeframe indicative of cancer survival [26].

Data analyses were performed in R version 4.0.4 [27]. Demographic and clinical characteristics were described by counselling status (counselled/not counselled) per study and for the total sample. Continuous variables were described using means and standard deviations (SD) and categorical variables with frequency of observations (n) and percentages (%). Differences between the two groups (counselled/not counselled) were examined on two primary outcomes i.e., objective knowledge of cancer genetics and genetic affinity, using *t*-test for means and chi-square or Fisher’s exact test for counts. The two-sided significance level was set at 5% for all tests, and Bonferroni corrections were used to address multiple testing.

A linear mixed-effect model examined factors that may influence the sum scores of primary outcomes, i.e., demographics, personal and family history of cancer, time since cancer diagnosis and time since genetic counselling, recruitment from genetic clinics or the cancer registry, and country (US and Switzerland). The mixed model incorporated a study-specific random intercept which accommodated for including subjects from the same family unit (non-independent observations) within each study. All factors were considered as fixed effects. To address factors influencing primary outcomes within family units, we also conducted sensitivity analyses by adding a family unit-specific random intercept to the previous linear mixed-effect model. The sensitivity analyses included only family units with more than one member enrolled in each of the three studies.

## 3. Results

The overall sample included a total of *n* = 1933 participants from *n* = 518 family units, with the majority (*n* = 1660, 85.9%) being from the US. Approximately 70% self-identified as White and 30% as belonging to minority racial or ethnic groups, i.e., Black or African American, American Indian or Alaskan Native, Arab or Arab American, Asian or Southeast Asian, Native Hawaiian or other Pacific Islander for the US-based samples; and African or Asian for the Swiss-based sample (Table 1). Given the small number of participants from minority racial and ethnic minority groups, we treated them as a single group in subsequent analyses.

Among participants, 745 (38.5%) had genetic counselling and 1188 (61.5%) did not. In the overall sample and in each individual study separately, participants who had counselling were more likely to have a cancer diagnosis compared to those who did not (69.5% vs. 50.6%, *p* < 0.0001). Those who had counselling were older, more likely to self-identify as White, married, and had higher education.

Knowledge of cancer genetics (total score) was overall higher in individuals who had counselling, with approximately 10 out of 13 items answered correctly (11, 9.5 and 9.5 items out of 13 in the three studies, respectively). The total score for individuals who did not have genetic counselling was 7.8 (Table 2).

The items least identified as risk factors in the overall sample, even among counselled individuals, were: “…is from Ashkenazi Jewish descent” (38.3% counselled and 13.5% not counselled), “having a relative diagnosed with breast cancer younger than 50 years old” (57.9% counselled and 63.6% not counselled “) and “…having a male relative with breast cancer” (65.1% counselled and 47.8% not counselled). All other items were answered correctly by more than 70% of participants who had counselling. “Having multiple relatives with breast cancer” was the one item identified as a genetic risk factor from more than 80% of all respondents (81.7% counselled and 80.6% not counselled).

Risk factors with the greatest discrepancies among individuals who had counselling and those who did not were: “…a family history of ovarian cancer” (74.6% counselled and 51.1% not counselled); “…a family history of breast cancer from the dad’s side of the family” (74.6% counselled and 56.7% not counselled); “…a pathogenic variant in the *BRCA1* or *BRCA2* genes” (88.1% counselled and 53.7% not counselled); and “…have cases of breast cancer in more than one generation” (84.6% counselled and 53.5% not counselled).

Individuals who had counselling reported higher genetic affinity and feeling more informed about the probability of getting cancer and about the genetics of cancer compared to those who did (Table 3). The total genetic affinity score was 7.3 out of 14 among those not counselled. There was a low-moderate correlation between knowledge of cancer genetics and genetic affinity in the overall sample (r = 0.38) and in the three studies (r = 0.28; r = 0.32; and r = 0.50, respectively).

Regression analyses in the overall sample showed that at the individual level higher genetic literacy (knowledge of cancer genetics and genetic affinity) were associated with having had counselling, less or equal to five years ago, a higher education, and a family history of cancer (Table 4). Being younger and self-identified as White were associated with higher knowledge of cancer genetics, while having had cancer was associated with higher genetic affinity. Sensitivity analysis at the family level, i.e., considering whether participants were members of the same family unit, showed that counselling, higher education, and a cancer diagnosis were still associated with higher knowledge of cancer genetics and with higher genetic affinity (Table 5). Younger age and self-identified as White were associated with higher knowledge of cancer genetics among members of the same family unit. Variance partition coefficients in sensitivity analysis showed that only 7% and 6% of variance in knowledge of cancer genetics and genetic affinity, respectively, was contributed by family clustering.

## 4. Discussion

This cross-study comparison used family-based data collected in the US and in Switzerland over a timeframe of more than 10 years to examine genetic literacy in individuals who had counselling for HBOC and their relatives who did not, and factors influencing genetic literacy both at the individual and at the family level. Genetic literacy was higher among participants who had counselling, compared to those who did not. Our findings support the role of genetic counseling in improving genetic literacy [1,3,18,19,28].

We identified specific risk factors and signs of HBOC that remain unclear, even to individuals who had a genetic consultation. Despite being important red flags for HBOC, early age of cancer onset, breast cancer in male relatives, and having Ashkenazi Jewish ancestry were not recognized as risk factors for most individuals. Genetic consultations provide personalized information and likely focus on individual risk factors. Thus, some of the above risk factors may not have been emphasized equally in all consultations, which may explain our findings. Nevertheless, HBOC cases need to be vigilant in identifying red flags in their family history since a new cancer diagnosis among relatives may provide important information that could change their own plans of managing hereditary cancer risk. Those who test negative (uninformative result) and those who do not qualify for testing are encouraged to periodically contact the genetic testing center and re-evaluate their status. Given the lifelong consequences of carrying an HBOC-associated pathogenic variant, periodic “check-ins” with genetic specialists can clarify important information and reassess cancer risk management plans.

Important risk factors, such as having a family history of ovarian cancer and a family history of breast cancer from the paternal side of the family were less frequently identified among individuals who did not have genetic counselling. This finding further highlights gaps in the dissemination of genetic information to at-risk individuals that have been reported over a period of 20 years [6,7,29,30,31]. Individuals who are unsure about how and from whom HBOC-associated pathogenic variants can be inherited are more likely to overlook their hereditary cancer risk if affected relatives are on the paternal side of the family. One possible explanation for this persistent finding may be related to unbalanced presentations of HBOC from mass media [32,33]. However, in light of the rapid evolution in cancer genetics, tracking changes in genetic literacy is extremely important. As knowledge continues to expand and educational materials are developed and made available to at-risk individuals and the lay public, the healthcare community needs to address these persistent knowledge gaps.

Consistent with studies that examined genetic literacy in the general population [3,6,8,9], participants who were younger, self-identified as White, had higher education, and a personal and/or a family history of cancer were more likely to know about risk factors and to feel better informed about cancer genetics. It is difficult to disentangle the effects of counselling from the experiential knowledge gained from a personal and/or a family history of cancer on genetic literacy. Our data show that having a consultation less than five years ago was associated with both higher knowledge of cancer genetics and higher genetic affinity, while time since a personal cancer diagnosis did not influence genetic literacy. These findings mean that the genetic consultation likely provides understandable and actionable information beyond the information that is discussed in the context of a personal cancer diagnosis [1,3,18,19,28].

Cascade testing for Tier 1 genetic conditions, such as HBOC, relies on assumptions of open family communication and effective dissemination of genetic information within members of family units. However, it is unclear if this communication strategy can ensure effective and accurate information transmission. We explored communication of genetic information within family units using sensitivity analysis, including only families with a member who received counseling and one or more at-risk relative who did not. By adding the random intercept term for each specific family unit into our modelling, unmeasured confounders, like level of family communication and information sharing between counselled and not counselled individuals, were controlled at that level. Interestingly, after adding family unit as a level in the analysis, genetic counselling was still significantly associated with knowledge of cancer genetics and with genetic affinity. Variance partition coefficients of sensitivity analyses showed that 6–7% of overall variation in objective knowledge and genetic affinity were explained by family clustering. If genetic information was openly and accurately shared from individuals who had counselling to their relatives, the variation in genetic literacy in members from different family units would have been observed more easily compared to the variation between members of random family units. This further implies that tailored educational interventions aiming to promote cascade testing should consider the characteristics of the family unit in addition to characteristics of the different individuals.

Using datasets from three studies could introduce a bias in the cross-study comparisons due to heterogeneity among the primary studies. In our case, the three primary studies had comparable aims and recruitment methods, which controlled for such bias and made comparisons feasible. Since participants from minority ethnic and racial groups had significantly lower levels of genetic literacy, our findings point to the widening gap of disparities in healthcare brought upon the clinical application of genetics [34,35,36]. However, participants from different ethnic and racial minority groups were very heterogeneous among the US and the Swiss-based samples, and were recruited primarily from one study. Thus, our findings are likely not applicable to non-White/Caucasian individuals and families, but without any inference to specific ethnic and racial minority groups. The Swiss sample was smaller, which may have also influenced findings regarding the impact of country and year of study on genetic literacy. HBOC status could only be ascertained for clinic-based samples. Finally, for the sensitivity analyses, we removed individuals without any relatives, which may have led to insufficient sample size.

## 5. Conclusions

Our cross-study comparison demonstrated the need for increased access to genetic information among at-risk individuals and that the lay public needs more assistance from healthcare professionals to understand complex genetic information and use it to inform plans for cancer risk management [37,38]. Our findings highlighted the role of counselling in improving genetic literacy and demonstrated persistent knowledge gaps and misconceptions, and that important red flags for HBOC remain poorly understood. Continued follow-up with genetic services could clarify and reinforce information that is overlooked or not well-understood. Addressing persistent knowledge gaps about aspects of HBOC, and racial and ethnic disparities in genetic care, should be priority public health goals. Efforts to improve family communication of genetic information should be enhanced with interventions at the clinical (support to carriers of pathogenic variants), legal (healthcare providers ability to provide tailored assistance with family communication) and public health (policies to improve access to genetic services) levels [14,39,40].

## Figures and Tables

**Table 1:** Demographics and clinical characteristics of the samples.

Characteristics	Total Sample *n* = 1933			Study 1 (2007) *n* = 370			Study 2 (2013) *n* = 1290			Study 3 (2017) *n* = 273		
	GC (+) * n = 745	GC (−) ^ *n* = 1188	*p*	GC (+) *n* = 200	GC (−) *n* = 170	*p*	GC (+) *n* = 313	GC (−) *n* = 977	*p*	GC (+) *n* = 232	GC (−) *n* = 41	*p*
Age (years)—mean (SD)	50.3 (10.3)	48.5 (11.0)	<0.001	50.6 (11.0)	48.7 (16.0)	0.53	48.7 (7.0)	48.3 (9.7)	0.53	52 (12.8)	51 (15.3)	0.70
Race and ethnicity—White (%)	78.4	69.5	<0.001	91.0	94.1	1	67.1	64.2	0.38	82.8	95.1	0.07
Married or Partnered—Yes (%)	86.7	93.9	<0.001	75.5	66.5	0.02	99.7	99.5	1	78.9	75.6	0.69
Elementary school (%)	10.3	20.9	<0.0001	8.5	14.1	0.04	15.7	22.7	0.001	4.7	4.9	0.79
High school degree (%)	50.1	56.9		24.5	31.2		62.3	61.4		55.6	56.1	
University/Post-graduate (%)	38.9	20.7		67.0	54.7		21.4	14.3		38.4	31.7	
Employed—Yes (%)	64.0	64.1	1	65.5	67.6	0.74	66.1	63.8	0.48	59.9	58.5	1
Cancer diagnosis—Yes (%)	69.5	50.6	<0.0001	53.5	11.8	<0.0001	89.7	59.2	<0.0001	56.0	7.3	<0.001
Family history cancer—Yes (%)	80.8	85.4	0.01	67.5	71.2	0.51	88.5	87.2	0.61	81.9	100.0	<0.01

* GC (+) Counselled; ^ GC (−) Not counselled.

**Table 2:** Objective knowledge of cancer genetics.

	Total Sample *n* = 1933			Study 1 (2007) *n* = 370			Study 2 (2013) *n* = 1290			Study 3 (2019) *n* = 273		
	GC (+) * n = 745	GC (−) ^ *n* = 1188		GC (+) *n* = 200	GC (−) *n* = 170		GC (+) *n* = 313	GC (−) *n* = 977		GC (+) *n* = 232	GC (−) *n* = 41	
	Correct (%)		*p*	Correct (%)		*p*	Correct (%)		*p*	Correct (%)		*p*
Cancer can be caused by a pathogenic variant passed on from one generation to the next	91.4	76.0	**<0.0001**	96.5	91.2	0.05	86.3	72.7	**<0.0001**	94.0	92.7	0.72
Families with a pathogenic variant in the *BRCA1* or *BRCA2* genes are likely to have cases of breast cancer in more than one generation	84.6	53.5	**<0.0001**	87.5	57.6	<0.001	77.6	51.4	**<0.0001**	91.4	87.8	0.55
A woman’s risk for getting breast cancer is higher when she…												
…has a family history of ovarian cancer	74.6	51.1	**<0.0001**	80.5	69.4	0.01	65.5	47.5	**<0.0001**	81.9	61.0	0.004
…has a relative diagnosed with breast cancer younger than 50 years old	57.9	63.6	0.01	72.0	61.8	0.04	76.7	66.1	**<0.001**	20.3	12.2	0.31
…has a family history of breast cancer from the dad’s side of the family	74.6	56.7	**<0.0001**	88.5	87.1	0.79	62.3	51.4	**<0.001**	79.3	58.5	<0.01
…has a family history of breast cancer from the mom’s side of the family	87.8	77.3	**<0.001**	93.5	92.9	0.99	82.7	75.1	**<0.01**	89.7	63.4	**<0.001**
…has breast and ovarian cancer in the same side of the family	82.0	68.7	**<0.0001**	88.0	85.3	0.54	78.9	66.6	**<0.0001**	81.0	48.8	**<0.001**
…has a pathogenic variant in the *BRCA1* or *BRCA2* genes	88.1	53.7	**<0.0001**	89.0	76.5	**<0.01**	82.1	49.0	**<0.0001**	95.3	61.0	**<0.0001**
…is from Ashkenazi Jewish descent	38.3	13.5	**<0.0001**	62.5	33.5	**<0.001**	32.2	10.3	**<0.0001**	25.4	4.9	<0.01
…has a male relative who had breast cancer	65.1	47.8	**<0.0001**	73.0	65.9	0.17	60.1	44.7	**<0.0001**	65.1	46.3	0.03
…has a relative with breast cancer in both breasts	78.9	68.3	**<0.001**	86.0	85.3	0.96	75.1	65.9	**<0.01**	78.0	53.7	**0.001**
…has a relative who had both breast and ovarian cancer	82.6	71.5	**<0.001**	85.0	84.1	0.92	81.5	69.8	**<0.0001**	81.9	61.0	<0.01
…has multiple relatives with breast cancer	81.7	80.6	0.58	91.5	94.1	0.44	84.7	79.7	0.06	69.4	46.3	<0.01
Total correct answers (0–13)—mean (SD)	9.9 (3.2)	7.8 (3.8)	**<0.0001**	10.9 (2.9)	9.8 (2.9)	**<0.001**	9.5 (3.6)	7.5 (3.8)	**<0.0001**	9.5 (2.8)	7.0 (3.9)	**0.0002**

* GC (+) Counselled; ^ GC (−) Not counselled. **Bold**: *p*-value still significant after Bonferroni correction.

**Table 3:** Genetic affinity.

	Total Sample *n* = 1933			Study 1 (2007) *n* = 370			Study 2 (2013) *n* = 1290			Study 3 (2019) *n* = 273		
	GC (+) * n = 745	GC (−) ^ *n* = 1188		GC (+) *n* = 200	GC (−) *n* = 170		GC (+) *n* = 313	GC (−) *n* = 977		GC (+) *n* = 232	GC (−) *n* = 41	
	Mean (SD)		*p*	Mean (SD)		*p*	Mean (SD)		*p*	Mean (SD)		*p*
How informed do you feel about the chances of getting cancer? (1–7)	5.7	4.7	**<0.0001**	6.1	4.9	**<0.0001**	5.5	4.6	**<0.0001**	5.7	4.9	0.02
	(1.3)	(1.8)		(1.2)	(1.4)		(1.6)	(1.8)		(1.1)	(1.8)	
How much do you know about the genetics of cancer? (1–7)	4.6	3.0	**<0.0001**	5.0	3.8	**<0.0001**	4.4	2.8	**<0.0001**	4.4	3.6	<0.01
	(1.5)	(1.7)		(1.2)	(1.6)		(1.7)	(1.6)		(1.4)	(1.7)	
Sum score (2–14)	10.0	7.3	**<0.0001**	10.9	8.6	**<0.0001**	9.5	7.1	**<0.0001**	9.9	8.1	**0.003**
	(2.9)	(3.3)		(2.4)	(2.8)		(3.4)	(3.3)		(2.3)	(3.6)	

* GC (+) Counselled; ^ GC (−) Not counselled. **Bold:** *p*-value still significant after Bonferroni correction.

**Table 4:** Fixed effects from linear mixed-effect models for factors influencing knowledge of cancer genetics and genetic affinity in the overall sample at the individual level.

	Knowledge of Cancer Genetics (*n* = 1895) *			Genetic Affinity (*n* = 1895) *		
	Estimate	Standard Error	*p*	Estimate	Standard Error	*p*
Age	−0.02	0.007	**<0.001**	−0.0004	0.007	0.95
Race and ethnicity (ref: White)	1.68	0.18	**<0.0001**	0.074	0.17	0.66
Education—(ref: Elementary school)	1.12	1.24	**<0.0001**	0.59	0.12	**<0.0001**
Employment (ref: No employment)	0.26	0.16	0.11	0.13	0.15	0.40
Cancer diagnosis (ref: No cancer)	0.21	0.21	0.33	0.59	0.21	**<0.01**
Genetic counselling (ref: No counselling)	0.80	0.27	**<0.01**	1.59	0.25	**<0.0001**
Family history of cancer (ref: No history)	1.45	0.25	**<0.0001**	0.50	0.23	**0.03**
Recruitment (ref: Clinic)	2.35	3.12	0.99	1.98	3.32	1.00
Country (ref: US)	2.82	3.13	0.99	1.38	3.32	1.00
≤5 years since cancer diagnosis (ref: Never diagnosed with cancer)	0.05	0.32	0.88	0.39	0.30	0.19
>5 years since cancer diagnosis (ref: Never diagnosed with cancer)	0.36	0.21	**0.09**	0.29	0.19	0.14
≤5 years since counselling (ref: Never counselled)	0.86	0.31	**<0.01**	0.34	0.29	0.21
>5 years since counselling (ref: Never counselled)	1.16	0.34	**<0.001**	0.68	0.32	**0.03**

* the number of participants is lower compared to the overall sample due to missing data. **Bold**: *p*-value still significant after Bonferroni correction.

**Table 5:** Fixed effects from linear mixed-effect model for factors influencing knowledge of cancer genetics and genetic affinity in members from the same family unit.

	Knowledge of Cancer Genetics (*n* = 1163) *			Genetic Affinity (*n* = 1163) *		
	Estimate	Standard Error	*p*	Estimate	Standard Error	*p*
Age	−0.03	0.008	**<0.0001**	<0.0001	0.007	0.99
Race and ethnicity (ref: White)	1.47	0.26	**<0.0001**	0.018	0.24	0.94
Education (ref: Elementary school)	0.98	0.15	**<0.0001**	0.54	0.14	**<0.0001**
Employment (ref: No employment)	0.27	0.20	0.18	−0.083	0.18	0.65
Cancer diagnosis (ref: No cancer)	0.72	0.27	**<0.01**	0.78	0.25	**0.002**
Genetic counselling (ref: No counselling)	0.84	0.32	**0.01**	1.63	0.30	**<0.0001**
Family history of cancer (ref: No history)	0.50	0.42	0.24	0.22	0.38	0.58
Recruitment (ref: Clinic)	1.81	1.80	0.24	1.86	2.21	0.40
Country (ref: US)	2.13	1.82	0.24	1.08	2.22	0.62
≤5 years since cancer diagnosis (ref: Never diagnosed with cancer)	−0.08	0.43	0.83	0.53	0.39	0.18
>5 years since cancer diagnosis (ref: Never diagnosed with cancer)	0.20	0.31	0.51	0.20	0.28	0.50
≤5 years since counselling (ref: Never counselled)	0.19	0.39	0.63	−0.03	0.35	0.93
>5 years since counselling (ref: Never counselled)	0.65	0.45	0.14	0.64	0.41	0.11

* the number of participants is lower compared to the overall sample. Individuals were members of 518 family units. **Bold**: *p*-value still significant after Bonferroni correction.

## Data Availability

Raw data are available upon request to Maria C. Katapodi (maria.katapodi@unibas.ch).
